# A native visual screening reporter-assisted CRISPR/Cas9 system for high-efficient genome editing in strawberry

**DOI:** 10.1186/s43897-025-00151-5

**Published:** 2025-06-03

**Authors:** Xianyan Han, Xia Liang, Dongdong Li, Miaoying Song, Zhimin Ma, Ruixia Li, Han Meng, Yue Cai, Bailong Song, Zhongchi Liu, Houcheng Zhou, Junhui Zhou

**Affiliations:** 1 Peking University Institute of Advanced Agricultural Sciences, Shandong Laboratory of Advanced Agricultural Sciences in Weifang, Shandong Provincial Key Laboratory of Precision Molecular Crop Design and Breeding, Shandong, 261325 China; 2 National Key Laboratory for Germplasm Innovation & Utilization of Horticultural Crops, Zhengzhou Fruit Research Institute, Chinese Academy of Agricultural Sciences, Zhengzhou, 450009 China; 3 College of Agriculture and Biotechnology, Zhejiang University, Zijingang Campus, Hangzhou, 310058 China; 4 Faculty of Synthetic Biology, Shenzhen University of Advanced Technology, Shenzhen, 518100 China; 5 Department of Cell Biology & Molecular Genetics, University of Maryland, College Park, MD 20742 USA; 6 National Engineering Research Center for Floriculture, Beijing Forestry University, Beijing, 100080 China

Genome editing has been extensively employed in gene functional studies and germplasm innovation in plants. However, its efficiency remains limited, particularly in species with complex chromosome compositions and long life cycles, such as fruit crops (Ma et al. 2023). Reporter genes derived from distantly related species, like GFP or *GUS*, often present challenges during tissue culture, including toxicity to tissue regeneration, susceptibility to integration into the plant genome, and difficulties in being removed. MYB10, a master transcription factor, regulates anthocyanin accumulation in strawberry plants (Medina-Puche et al. 2014). *RAP* and *RAP-L2*, which encode a glutathione S-transferase, mediate anthocyanin transportation from the cytosol to the vacuole, resulting in red petioles (Luo et al. 2018). These red-pigment promoting genes serve as promising candidates for native reporters in selecting successful transgenic events during transformation.

In this study, we developed an endogenous calli-specific promoter-driven red pigment reporter system for a highly efficient, straightforward, and labor-saving screening strategy for transgenic lines, termed Native Visual Screening Reporter (NVSR). The integration of NVSR into our CRISPR/Cas9 construct facilitates simple identification of Cas9-positive transgenic lines in the T_0_ generation and Cas9-free seedlings in subsequent generations.

*Fragaria vesca* (*F. vesca*), a wild diploid strawberry variety, serves as a model plant in this research. To determine the most effective red-color reporter in the calli stage, the aforementioned three red candidate reporter genes were over-expressed through *Agrobacterium*-mediated transformation (Zhou et al. 2018). Only *FveMYB10 *overexpression (*FveMYB10*-OE) induced a visible red color in the newly generated calli, while *FveRAP*-OE and *FveRAP-L2*-OE failed to produce red pigment accumulation (Fig. 1A, B; Supplementary Table S1). Consequently, *FveMYB10* was chosen for subsequent investigation.

**Fig. 1:**
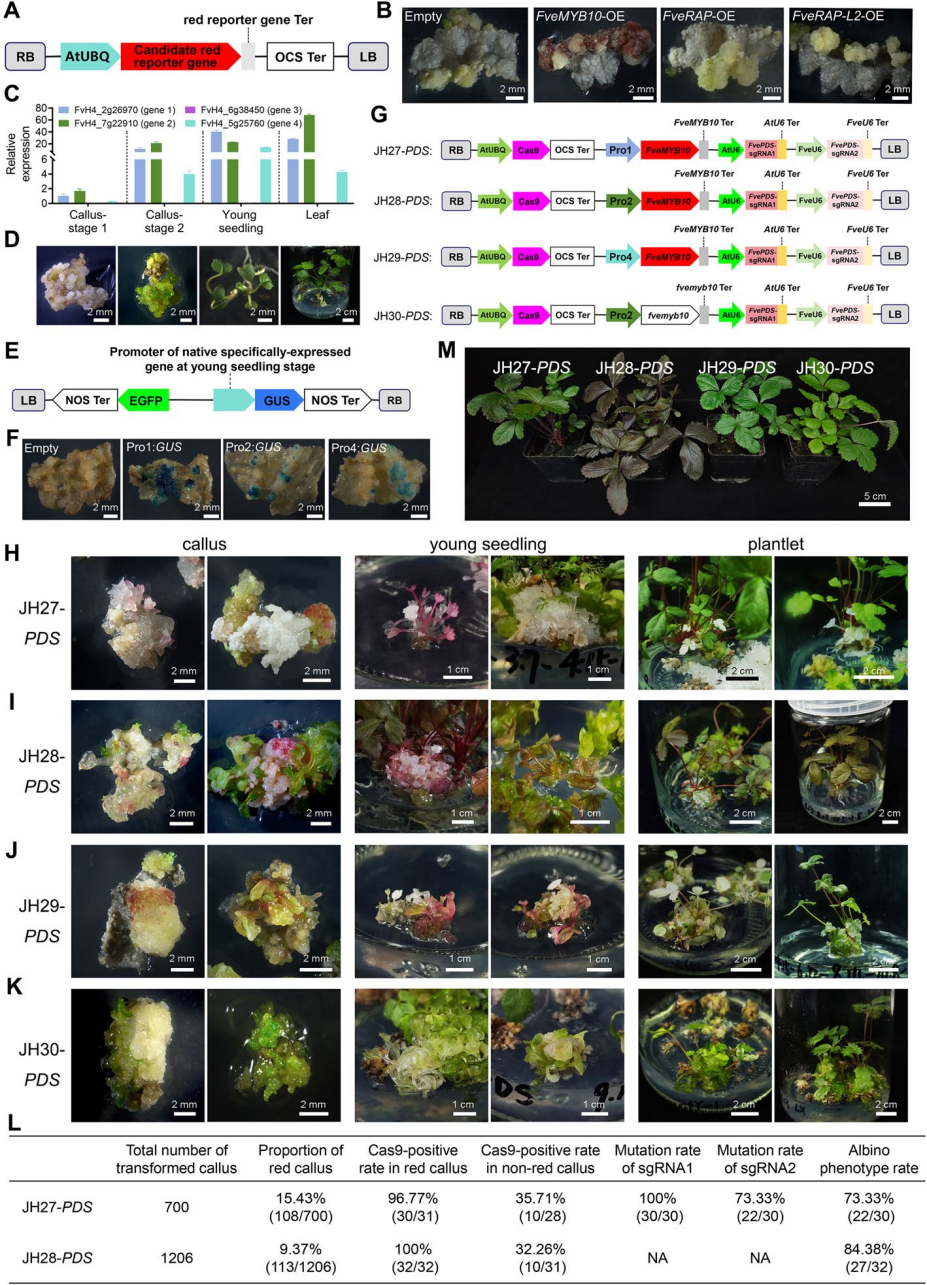
High-efficiency genome editing in strawberry using the NVSR-assisted CRISPR/Cas9 systems. **A** Schematic illustration of OE-red reporter vector. The candidate red reporter genes were driven by AtUBQ10 promoter. **B** Phenotypes of transgenic *F. vesca* calli expressing empty vector, *FveMYB10-*OE, *FveRAP-*OE, and *FveRAP-L2-*OE constructs. Images were captured two months post-*F. vesca* calli transformation. **C** Relative expression levels of candidate tissue-specific genes at different stages. **D** Graphical illustration of four growth stages during strawberry transformation. Early-stage calli without shoot appearance were classified as callus stage 1, while later-stage calli with emerged shoots were classified as callus stage 2. **E** Schematic illustration of the *GUS*-containing construct driven by candidate calli-specific promoters. **F** *GUS* staining results of *F. vesca* calli expressing *GUS* reporters. **G** Schematic illustration of the CRISPR constructs (JH27-*PDS*, JH28-*PDS*, JH29-*PDS*, and JH30-*PDS*) containing native AtUBQ:Cas9-Ter^ocs^-Pro:*FveMYB10*-Ter^MYB10^-sgRNA(PDS). **H–K** Phenotypes of transgenic lines at different stages (callus, young seedling, and plantlet) of JH27-*PDS*, JH28-*PDS*, JH29-*PDS*, and JH30-*PDS*. **L** Summary of transgenic efficiency and genome editing efficiency. NA: not sequenced. **M** Phenotypes of transgenic plants expressing JH27-*PDS*, JH28-*PDS*, JH29-*PDS*, and JH30-*PDS* in mature plantlets. Scale bars are shown in the figure

Based on a previously developed RNA-seq database (Kang et al. 2013), we conducted a genome-wide screening to identify candidate promoters with specific expression in young seedlings or callus tissues. Four candidate genes, referred to as candidate genes 1 − 4, were selected based on their expression profiles (Fig. S1; Supplementary Table S2). To validate their tissue-specific expression, we performed quantitative real-time PCR (qRT-PCR) and found that three of the four genes exhibited expression patterns consistent with the RNA-seq data (Fig. 1C, D; Supplementary Table S1). The promoter regions of genes 1 − 4, designated as Pro1 − Pro4, were amplified from *F. vesca* genomic DNA (gDNA) and fused to a *GUS* reporter gene, respectively (Fig. 1E; Supplementary Table S1). Subsequently, *Agrobacterium* strains containing Pro1:*GUS*, Pro2:*GUS*, Pro3:*GUS*, and Pro4:*GUS* constructs were infiltrated into tobacco leaves and transformed into *F. vesca* calli. Strong blue *GUS* signals were detected in the infiltrated tobacco leaves (Fig. S2) and transformed *F. vesca* calli (Fig. 1F) for Pro1, Pro2, and Pro4 constructs, while no noticeable *GUS* signal was observed for the Pro3 construct. Consequently, Pro1, Pro2, and Pro4 were selected for further investigation.

To integrate our NVSR into CRISPR vectors, we developed three expression cassettes: Pro1:*FveMYB10*-Ter^MYB10^, Pro2:*FveMYB10*-Ter^MYB10^, and Pro4:*FveMYB10*-Ter^MYB10^. These cassettes replaced the original 35S:GFP-Ter^OCS^ in the CRISPR/Cas9 construct JH19 (Zhou et al. 2018), resulting in three new constructs: AtUBQ:Cas9-Pro1:*FveMYB10*-Ter^MYB10^ (JH27), AtUBQ:Cas9-Pro2:*FveMYB10*-Ter^MYB10^ (JH28), and AtUBQ:Cas9-Pro4:*FveMYB10*-Ter^MYB10^ (JH29). Additionally, we amplified *fvemyb10*, the *FveMYB10* mutant caused by an SNP in the white-fruit phenotype of YW5AF7 (Hawkins et al. 2016), and incorporated it into JH19, creating the control construct AtUBQ:Cas9-Pro2:*fvemyb10*-Ter^myb10^ (JH30) (Fig. 1G; Fig. S3; Supplementary Table S1).

*Phytoene Desaturase* (*PDS*) was selected as the target gene for NVSR-CRISPR editing and incorporated into JH27 to JH30. Following Agro-infiltration, red pigmentation was observed at the edges of calli transformed with JH27-*PDS*, JH28-*PDS*, and JH29-*PDS* but not with JH30-*PDS* (Fig. 1H-K). A more intense red coloration was evident at the early young seedling stage of JH27-*PDS* and JH29-*PDS*, which gradually diminished at later stages, coinciding eventually giving way to an albino phenotype (Fig. 1H, J). Red pigments in JH28-*PDS* leaves and petioles also accumulated at earlier seedling stages and remained detectable at later seedling and plantlet stages. This finding indicates that Pro2 retains activity, albeit at reduced levels, potentially influencing plant development. In contrast to JH28-*PDS*, JH30-*PDS* induced only the albino phenotype without producing red pigments. Notably, over 70% of the red calli ultimately developed the albino phenotype, suggesting successful editing of the target gene *FvePDS*.

To assess the transgenic status of the red calli, we randomly selected 31 red calli and 28 non-red calli expressing JH27-*PDS* for Sanger sequencing analysis. The results revealed that approximately 97% (30 out of 31) of the red calli were Cas9-positive, while only 36% (10 out of 28) of the non-red calli tested positive for Cas9 (Fig. 1L; Fig. S4A-B; Supplementary Table S1). Similarly, for JH28-*PDS*, 100% (32/32) of the red calli were both Cas9-positive (Fig. S4C-D) and *FveMYB10*-positive (Fig. S5), indicating a strong correlation between stable transformation and the expression of the red-colored reporter.

To evaluate whether *FveMYB10* reporters influence editing efficiency, we examined *FvePDS* editing of sgRNA 1 and sgRNA 2 in 30 red calli transformed with JH27-*PDS*, identifying various mutation types (Fig. S6; Supplementary Table S1 and S2). The mutation rates for sgRNA 1 and sgRNA 2 were 100% and 73.3%, respectively, which align with the editing efficiency (49%-75%) reported in our previous study (Zhou et al. 2018). Notably, 73%-84% of Cas9-positive lines displayed an albino phenotype (Fig. 1L), and no off-target effects were observed (Fig. S7; Supplementary Table S1). These findings indicate that the incorporation of the *FveMYB10* reporter does not adversely affect editing efficiency.

To evaluate the potential impact of the *FveMYB10* reporter on growth in transgenic lines, we transferred the transgenic plants to soil and monitored their development. The JH28-*PDS* construct induced a red coloration throughout the strawberry plants, including leaves and flowers. In contrast, constructs JH27-*PDS* and JH29-*PDS* only resulted in a mild red hue on young leaves, with no observable effect on mature leaves and flowers (Fig. 1M; Fig. S8). These observations suggest minimal interference with morphogenesis and development during the later stages of strawberry growth. Total anthocyanin content in these transgenic lines was quantified following previously established methods (Luo et al. 2018). Co-expression of NVSR (JH27 and JH29) demonstrated limited impact on anthocyanin accumulation in leaves (Fig. S9). Overall, the Pro1 and Pro4-driven *FveMYB10* reporter systems (JH27 and JH29) appear to be suitable reporters for identifying transgenic positive strawberry lines in T_0_ plants without apparent side effects. Furthermore, the Pro2-driven *FveMYB10* reporter system (JH28) could serve as a visual indicator to differentiate between Cas9-free lines and Cas9 lines in the T_1_ generation, as the red-colored reporter and Cas9 are genetically linked (Fig. S10).

In this study, our *FveMYB10* reporter is driven by a native tissue-specific promoter and terminator, distinguishing it from other reporters such as GFP, *GUS*, or *RUBY* (He et al. 2020), as it comprises all components from the strawberry's endogenous genome, avoiding the removal of exogenous fragments via backcross separation after conventional genetic transformation, especially for crops with high heterozygosity such as strawberries. This novel reporter was shown to be easily scorable by naked eyes and enables rapid screening for transgenic positive strawberry lines. Moreover, the promoter driving *FveMYB10* is temporally regulated in JH27 and JH29; the red pigments initially indicative of transgene-positive calli subsequently disappear at later stages, exerting minimal impact on plant transformation efficiency or development. Our NVSR reporter can be readily eliminated in the next generation along with Cas9 during segregation. Furthermore, our NVSR system exhibits high sensitivity in identifying transgene-positive lines without requiring specialized fluorescence equipment or costly substrates during selection, thus providing a series of reliable, cost-effective, and convenient reporters for strawberry transformation and genome editing. Additionally, we applied our JH27 and JH28 reporters in raspberry transformation, observing similar red pigments in the calli (Fig. S11). Given that MYB10 or MYB10-like transcription factors play a conserved role in anthocyanin biosynthesis, leading to anthocyanin accumulation in many plants (Allan et al. 2008; Chagné et al. 2013; Holušová et al. 2022; Liu et al. 2021; Wang et al. 2022), this strategy may be highly adaptable to other crop plants.

## Supplementary Information

Supplementary Material 1.

Supplementary Material 2.

Supplementary Material 3.

## Data Availability

The datasets utilized and analyzed in this study are accessible within the article and its accompanying online supplementary material.
