# Supplementary material for: A native visual screening reporter-assisted CRISPR/Cas9 system for high-efficient genome editing in strawberry
Source: Mol Hortic. 2025 Jun 3;5:29. doi: 10.1186/s43897-025-00151-5 (PMC12131447; doi:10.1186/s43897-025-00151-5)
Supplement: Supplementary file 3 — Supplementary Material 3. [file 43897_2025_151_MOESM3_ESM.docx]

**A native visual screening reporter-assisted CRISPR/Cas9 system for high-efficient genome editing in strawberry**

Xianyan Han^1,2,^^†^, Xia Liang^1,6†^, Dongdong Li^3^, Miaoying Song^1^, Zhimin Ma^1^, Ruixia Li^1^, Han Meng^1^, Yue Cai^1^, Bailong Song^1^, Zhongchi Liu^4,5^, Houcheng Zhou^2,*^ and Junhui Zhou^1,*^

^1^ Peking University Institute of Advanced Agricultural Sciences, Shandong Laboratory of Advanced Agricultural Sciences in Weifang, Shandong Provincial Key Laboratory of Precision Molecular Crop Design and Breeding, Shandong 261325, China

^2^ National Key Laboratory for Germplasm Innovation & Utilization of Horticultural Crops, Zhengzhou Fruit Research Institute, Chinese Academy of Agricultural Sciences, Zhengzhou 450009, China

^3^ College of Agriculture and Biotechnology, Zhejiang University, Zijingang Campus, Hangzhou 310058, China

^4^ Faculty of Synthetic Biology, Shenzhen University of Advanced Technology, Shenzhen 518100, China

^5^ Department of Cell Biology & Molecular Genetics, University of Maryland, College Park, MD 20742, United States

^6^ National Engineering Research Center for Floriculture, Beijing Forestry University, Beijing 100080, China

^*^ Correspondence: [junhui.zhou@pku-iaas.edu.cn](mailto:junhui.zhou@pku-iaas.edu.cn) or [zhouhoucheng@caas.cn](mailto:zhouhoucheng@caas.cn)

^†^ These authors contributed equally to this work.

**Keywords:** Strawberry, *FveMYB10*, Visual reporter, Transformation, CRISPR/Cas9


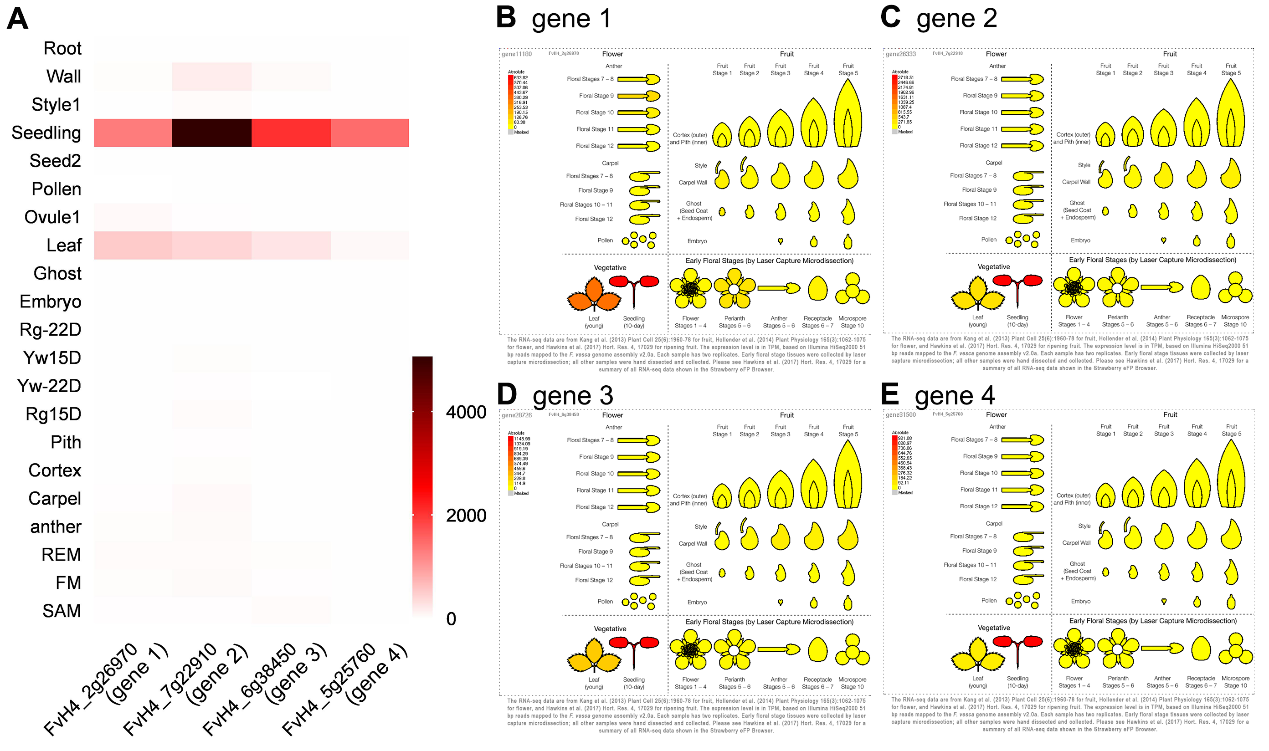


**Supplementary Fig. S1.** Expression patterns of candidate tissue-specifically expressed genes of YW5AF7. **A** Heatmap of four tissue-specifically expressed genes at young-seedling stage screened from RNA-seq data. **B-E** Predictions through the Bar website of four tissue-specific expressed genes at the young-seedling stage of YW5AF7 plant. **B** FvH4_2g26970 (gene1); **C** FvH4_7g22910 (gene2); **D** FvH4_6g38450 (gene3); **E** FvH4_5g25760 (gene4).


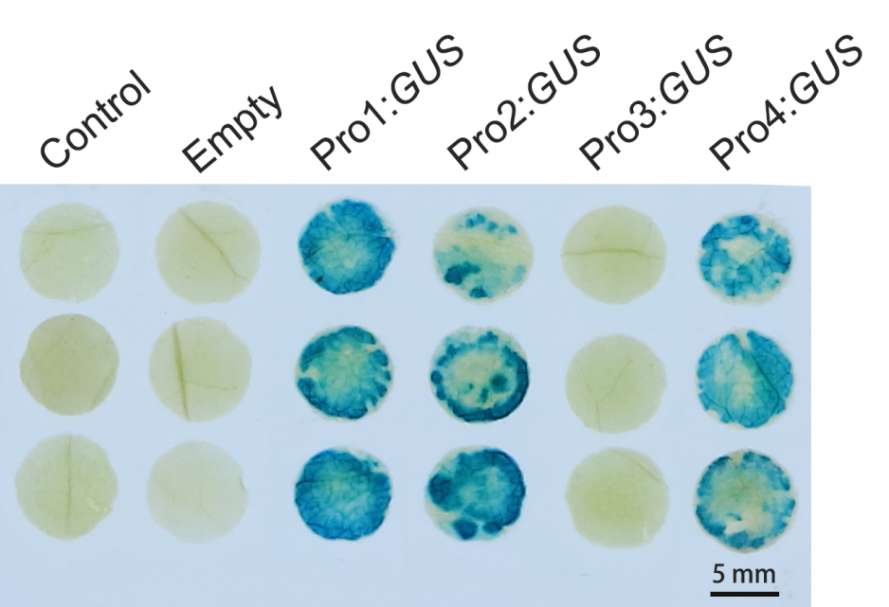


**Supplementary Fig. S2.** *GUS* staining of tobacco leaves infiltrated with *Agrobacterium* strains containing *GUS* reporter. Control: tobacco leaves without expression vectors; Empty: tobacco leaves containing DX2181G-*GUS* empty vector (with no promoters). Scale bars were shown in the figure.

**
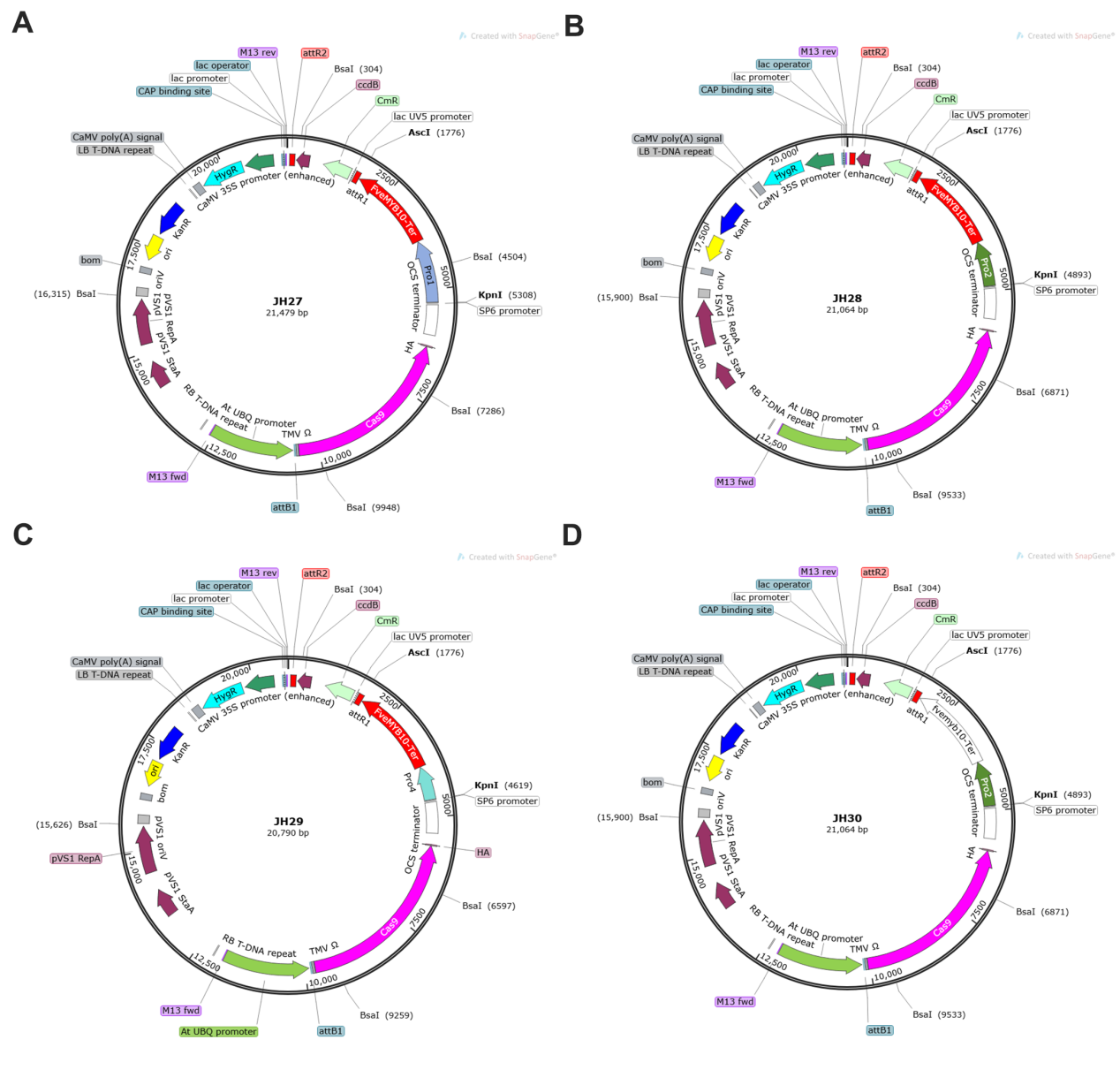
**

**Supplementary Fig. S3.** Schematic illustration of the NVSR-CRISPR vectors **A** JH27, **B** JH28, **C** JH29 and **D** JH30.


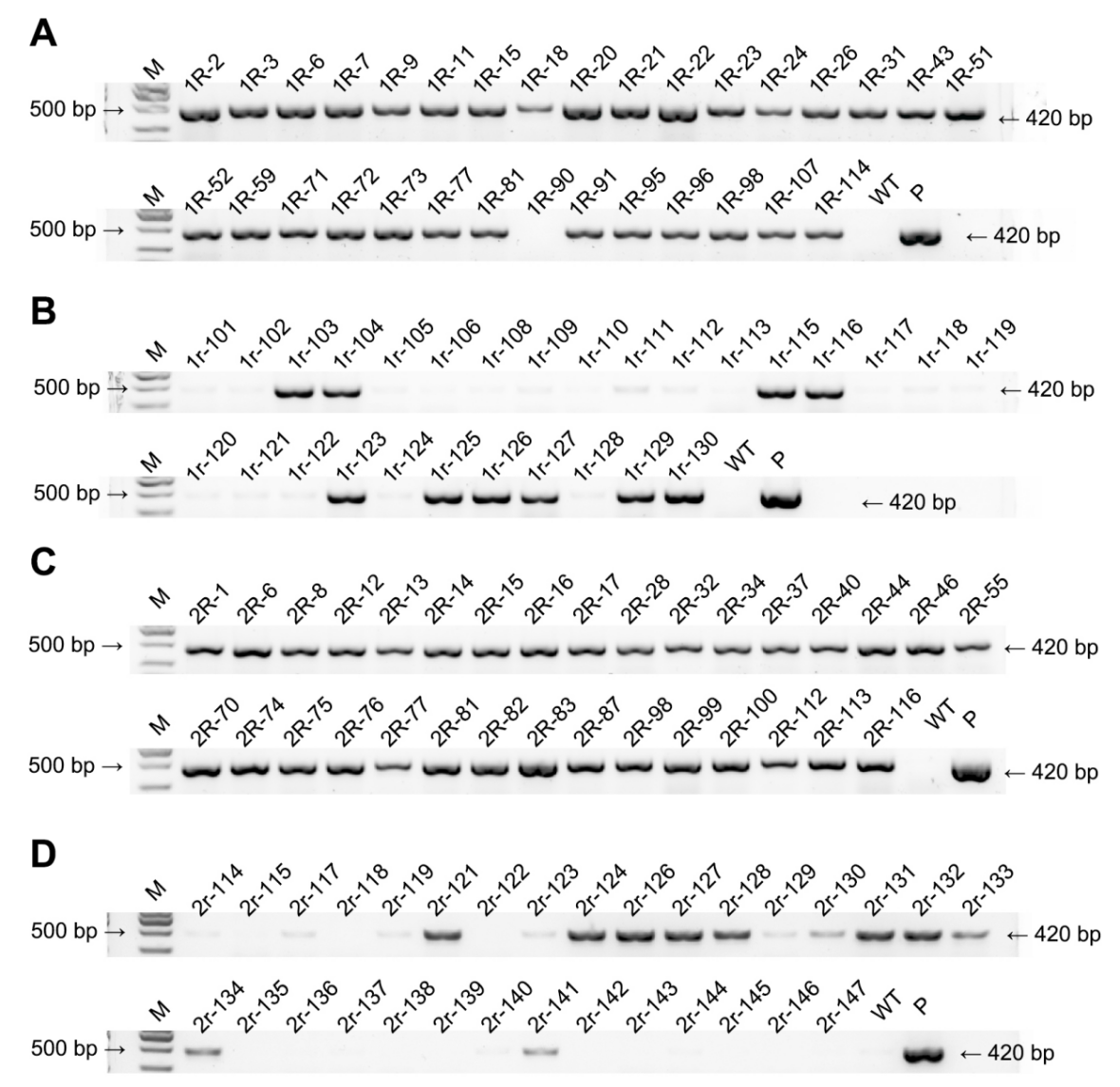


**Supplementary Fig. S4.** Cas9-positive detection of **A-B** JH27-*PDS* and **C-D** JH28*-PDS* transgenic calli. M, DNA marker; WT, wild type as a negative control; P, the corresponding plasmid as a positive control. The capital letter ‘R’ stands for a red callus, while the small letter ‘r’ represents a non-red callus.


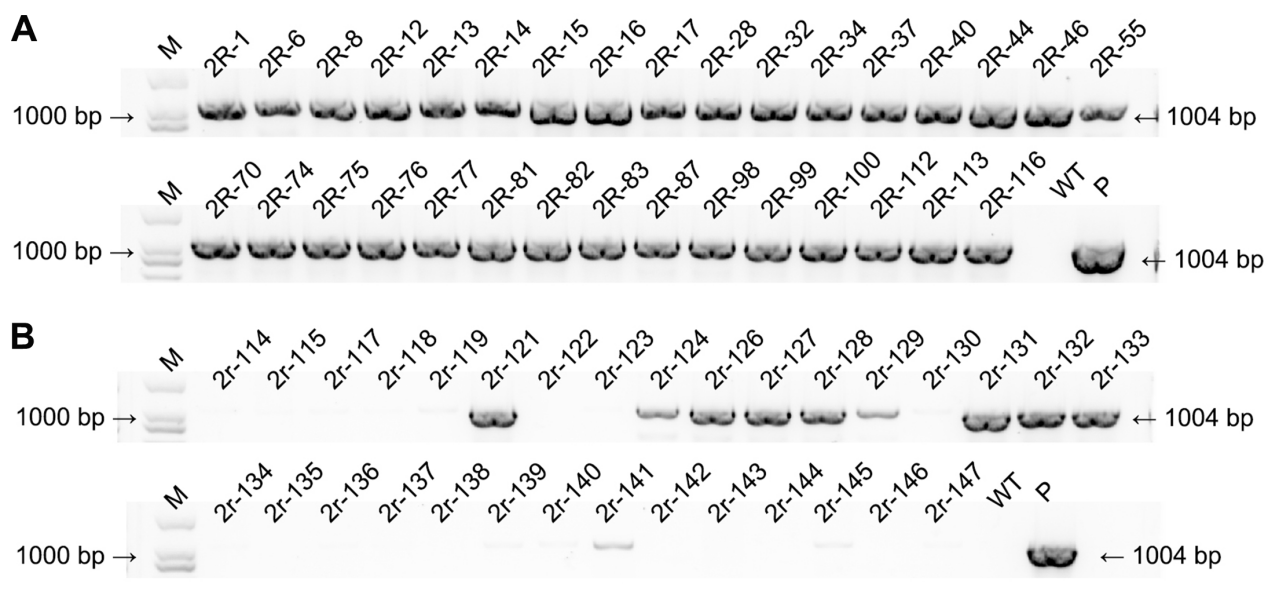


**Supplementary Fig. S5.** PCR amplification of *FveMYB10* fragment in **A** red and **B** non-red calli in transgenic plants expressing JH28*-PDS*. M, DNA marker; WT, wild type as a negative control; P, the corresponding plasmid as a positive control. The capital letter ‘R’ stands for a red callus, while the small letter ‘r’ represents a non-red callus.


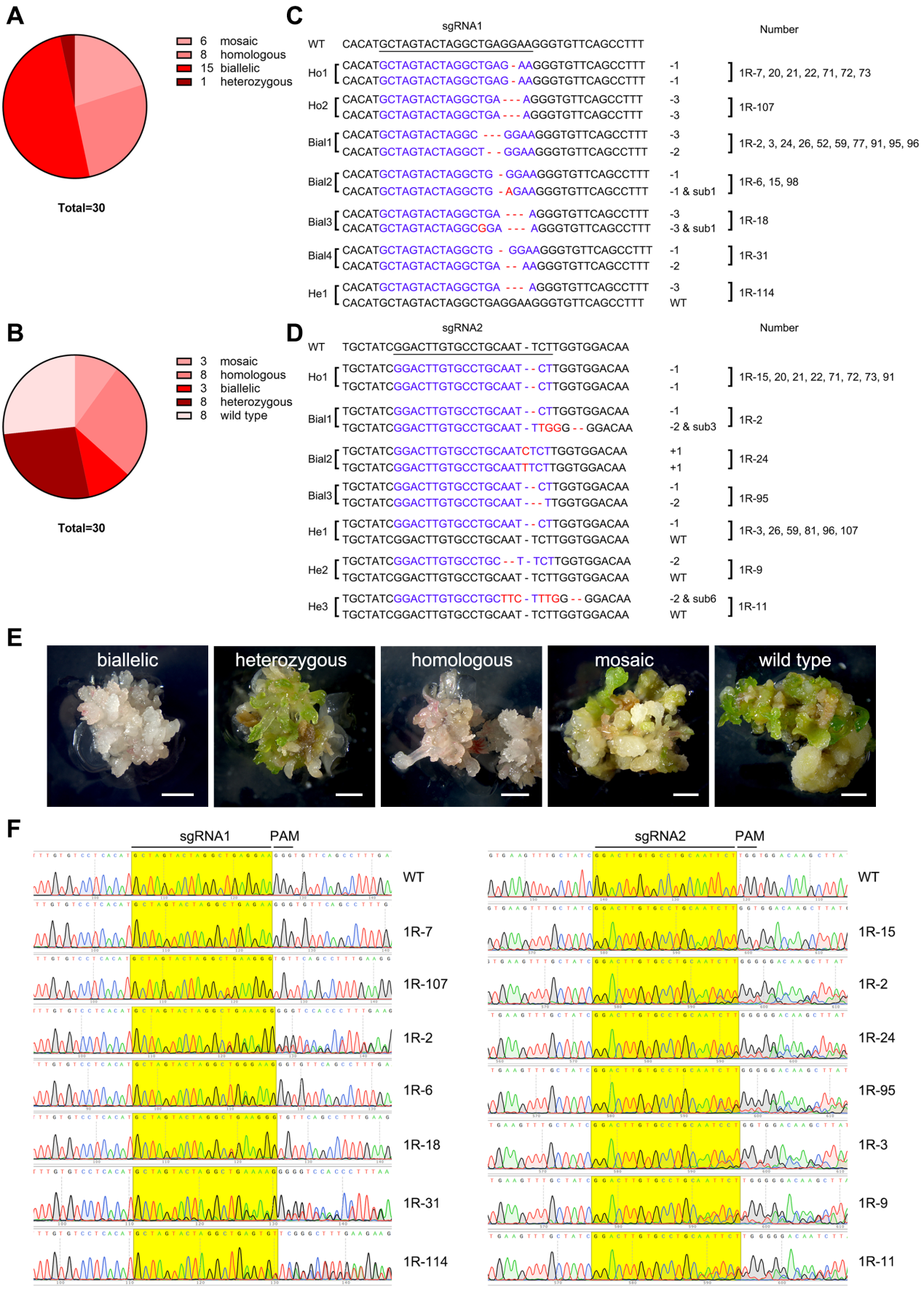


**Supplementary Fig. S6.** Mutation detection of *FvePDS* in JH27-*PDS* transgenic materials. **A** Pie chart of gene editing types for sgRNA 1. **B** Pie chart of gene editing types for sgRNA 2. **C-D** Gene editing types of sgRNA 1 (**C**) and sgRNA 2 (**D**). WT, wild type; Ho, homologous; Bial, biallelic; He, heterozygous. **E** Phenotypes of different gene editing types. Scale bars: 2 mm. **F** Targeted mutagenesis of *FvePDS* and Sanger sequencing chromatograms of the resulting JH27-*PDS* transgenic lines.


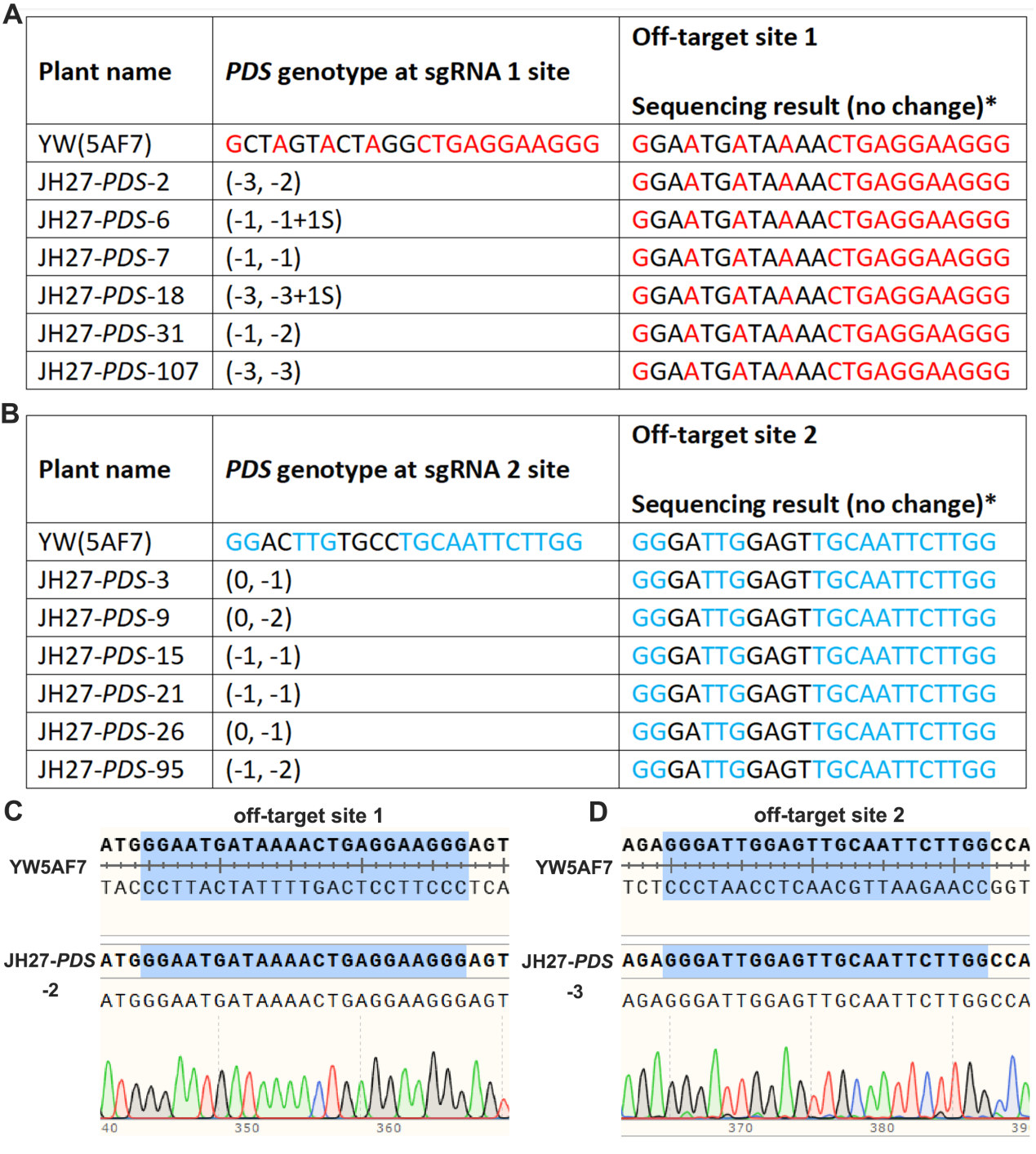


**Supplementary Fig. S7.** Summary of off-target site sequencing results for T_0_ generation JH27-*PDS* transgenic plants. **A** Red nucleotides are homologous between *FvePDS* sgRNA 1 site and the off-target site 1. **B** Blue nucleotides are homologous between *FvePDS* sgRNA 2 site and the off-target site 2. ‘-’ sign stands for deletion, 0 indicates no change and 1S indicates single base substitution. **C-D** Sanger sequencing chromatograms of off-target site 1 and off-target site 2 for JH27-*PDS* transgenic line.


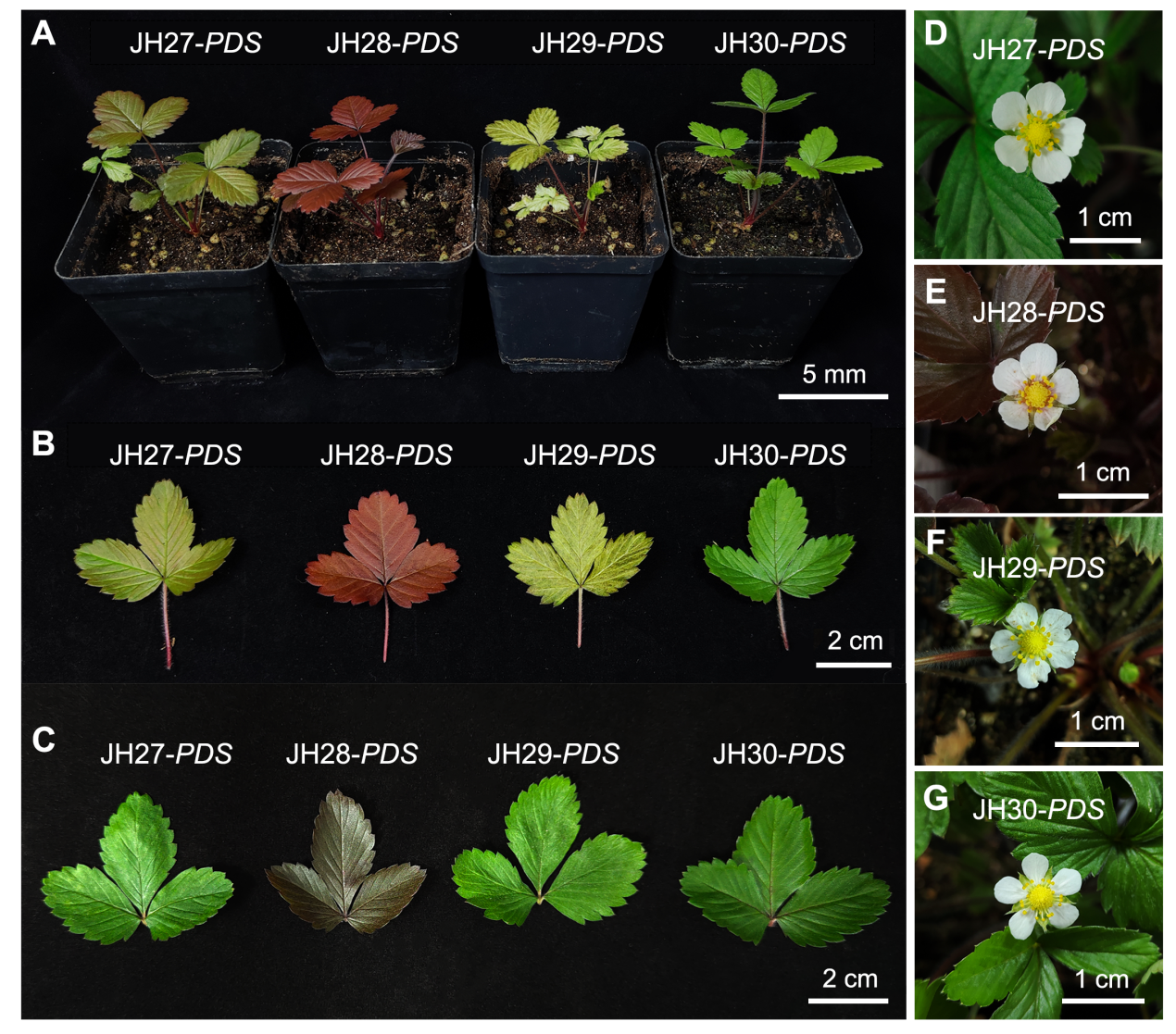


**Supplementary Fig. S8.** Phenotypes of transgenic plants expressing JH27-*PDS*, JH28-*PDS*, JH29-*PDS*, and JH30-*PDS* in **A** young plantlet, **B** young leaves, **C** old leaves and **D-G** flowers. Scale bars were shown in the figure.

**
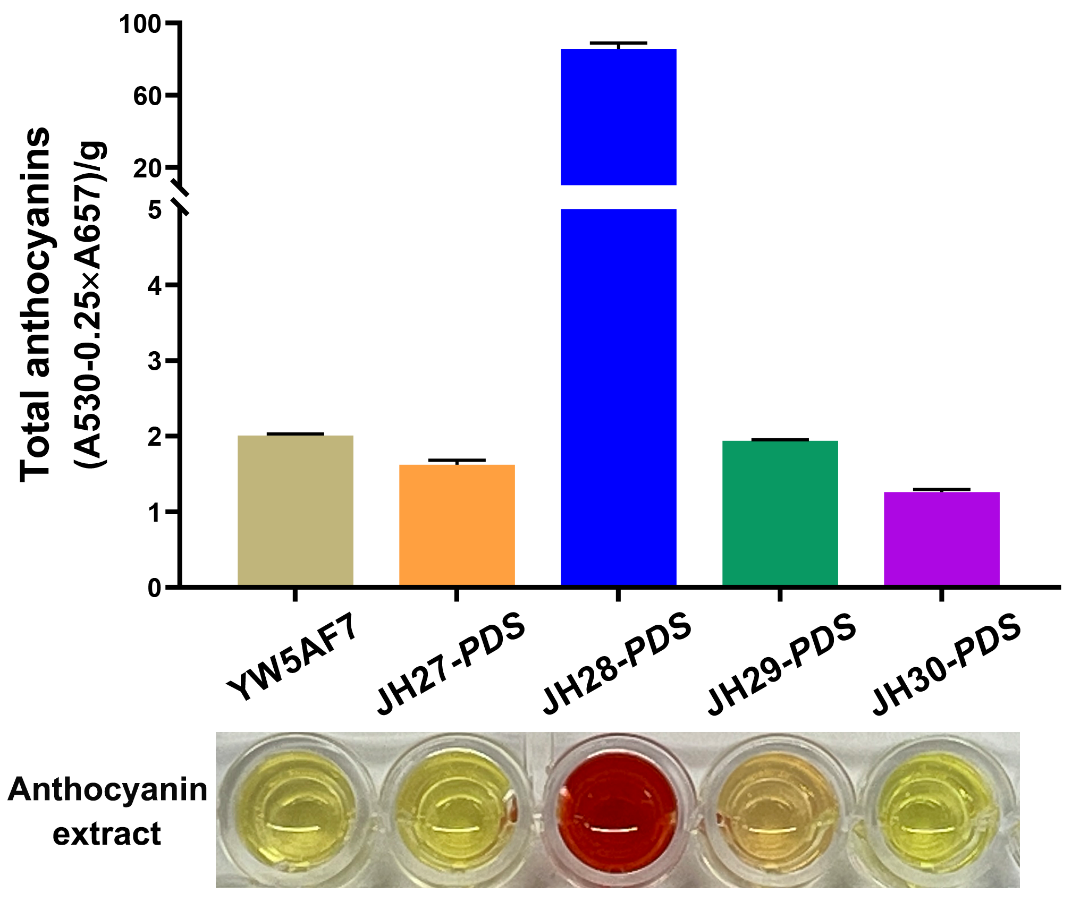
**

**Supplementary Fig. S9.** The total anthocyanin contents in the leaves of JH27-*PDS*, JH28-*PDS*, JH29-*PDS* and JH30-*PDS* transgenic plants. Wild type of YW5AF7 plants were used as control. Data are means ± SD obtained from three biological replicates.

**
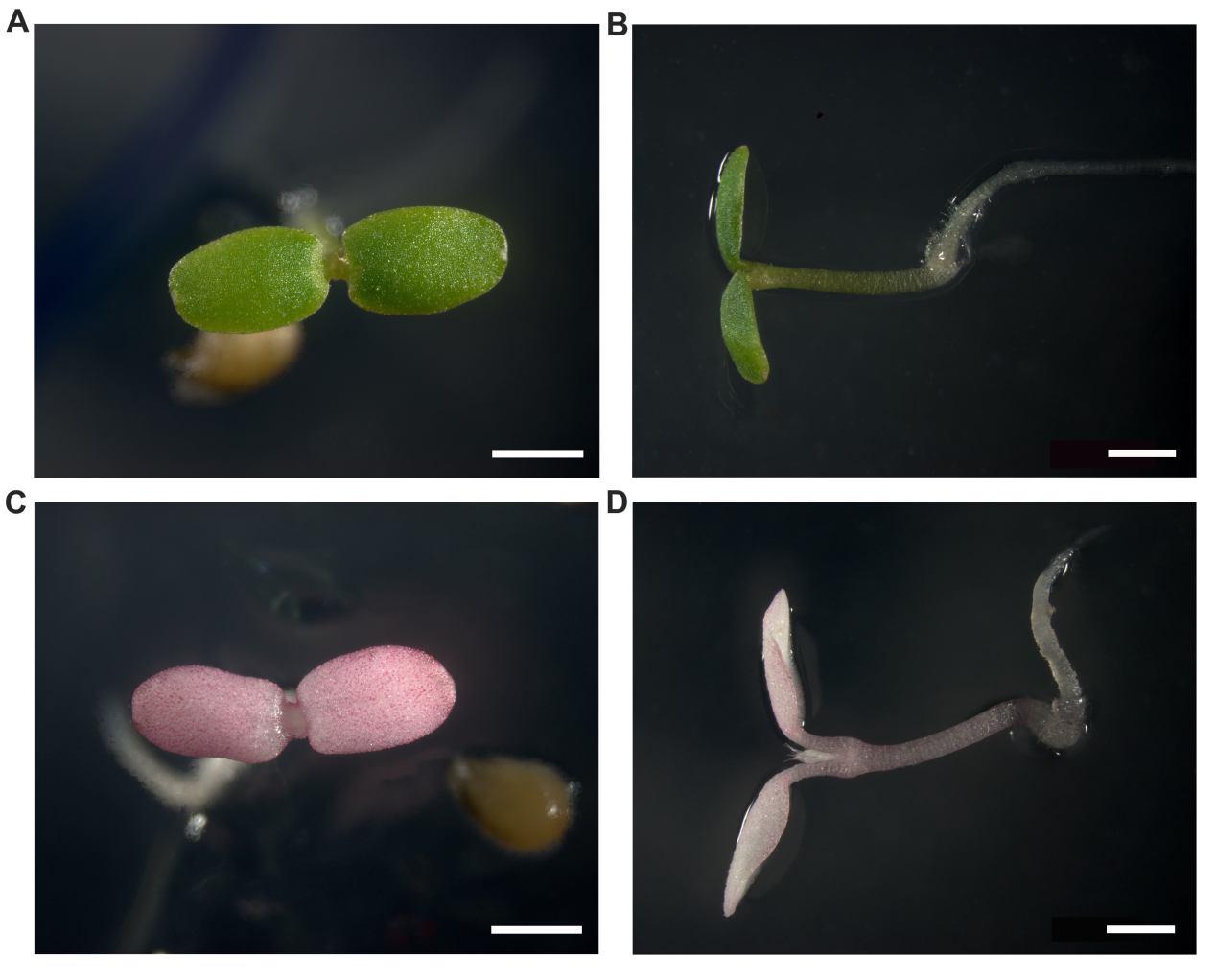
**

**Supplementary Fig. S10.** Phenotypes of T_1_ transgenic plants of JH27-*PDS* at young seedling stage. **A-B** Wild type of YW5AF7 plant as control; **C-D** T_1_ transgenic plants of JH27-*PDS*. Scale bars: 1 mm.


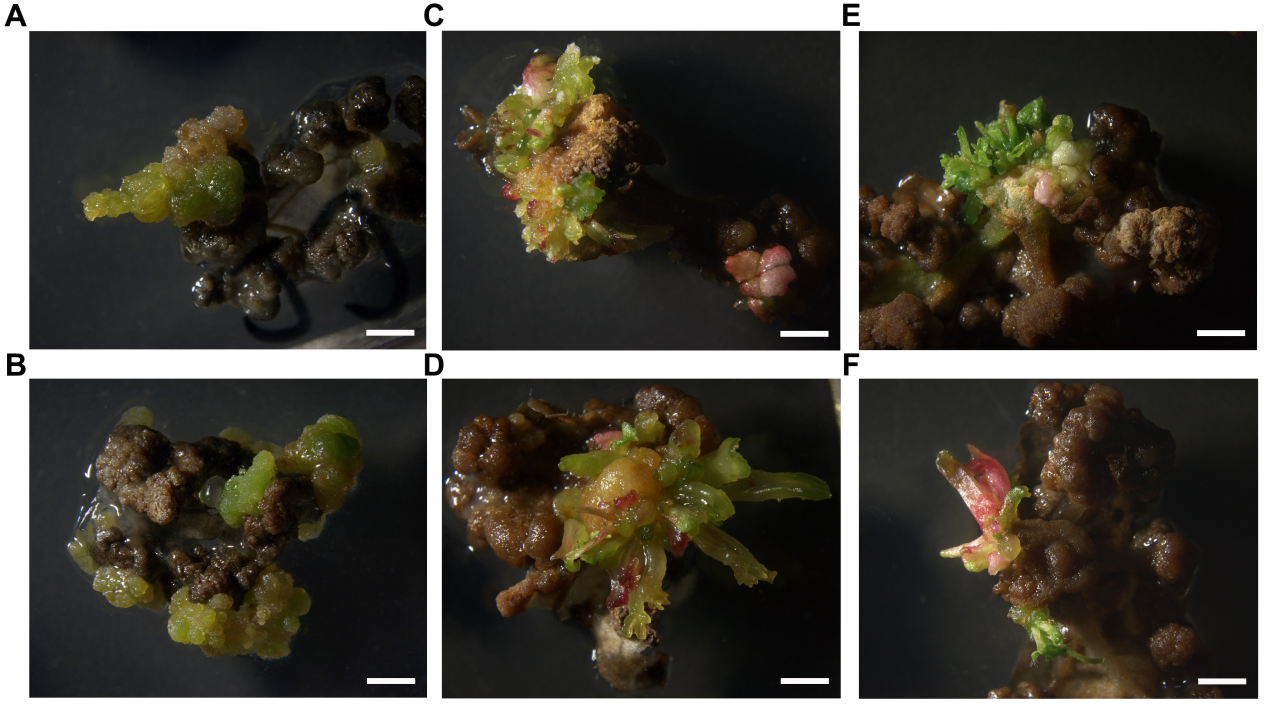


**Supplementary Fig. S11.** Phenotypes of *Rubus idaeus* ‘Joan J’ transgenic calli expressing **A-B** JH19 vector, **C-D** JH27-*PDS* and **E-F** JH28-*PDS* constructs. Scale bars: 2 mm.

**Appendix S1. A 2046bp fragment (*FveMYB10-*Ter^MYB10^) across *FveMYB10* genomic DNA and terminator of red-fruit accession Rüegen.**

ATGGAGGGTTATTTCGGTGTGAGAAAAGGTGCATGGACTAAAGAGGAAGATGAACTTCTGAAACAGTTCATCGAAATTCATGGAGAAGGCAAATGGCATCATGTTCCTCTCAAATCAGGTAGTTAATCTAAGATTACGTACATGCTCTCACTGGAAAGTTTAGACTGCTGCTTATATGTATGTTTACGTCTGTACTTGCAGGCTTAAACAGATGCAGGAAGAGCTGTAGACTGAGATGGCTGAATTATTTGAAGCCGAATATCAAGAGAGGAGAGTTTGCAGAGGATGAAGTTGATTTGATCATCAGGCTTCATAAGCTTCTAGGAAACAGGTAATTAGAGCATCTGATATGTTCGTTTCTGCTTAATTTCTCAGTTACTGTTCTCTCAGGTAAAGCATGCGTTATTTCTTCTGGAATTCCAAAGCTAACTATATATATATATATATATATATATATATATTTACATATGTTTTCCTTGAATAATAAATATCTAGAGTGGTGGTCATTTCTGCTACTTTTACGCTTTTAATTAGCCCATGCATCCTCCTTTCATATCATGCGTACATTCCTTCATACAATCATACCCACTTACTTTCAGTGTAGGGAACTCCTTTAGCTGAAGTATTGGTGCTTATTATTGCCTGATGGCTACGGCTTATGCTTGCCTAGCTAGCTATCCATGAACTAAACATCAAAATGTCTACATAGCCTTTTAACGAAATTTATCATGAATTAATTACAAATTATTTGAACTAAAAAGATTCTTTTATAATTTCATAACTGACTGACAGATGTTTATATATAATATATATACACGTGTGATGTGAAGAACTATTGGAATATATAATATAGTGAGGATTTTGCTACTGTGTTTGATGCACGCTAAATTAAAACAAAAGAAGACAAATGTATGATCTTGTTCTTCTTCTTGTAATCCGTAAAAATTGGATTTTGTTTTCCAGATTTTAAAATGTGATTTTTCTTTCTTTTCTTTTAATTTGATTTGTTATTAAATTAGGTAATATCACGTCACTTGGCACATCAATTAGTAATATGTTGACATCATATTTAGGATTGATCTAGACCAATCATTGTTTTAAAATCTAATGGCTAACAATTAGTGTCAAAATTTGTGTCAAATTTAATGTCCCTTAATGTCCCTTGAACCGGACCCTATATGACATACATTTAATATTGTAGAAATTGCCTTGCATCTGTTAAGTTTCTAATGTGTTTCTTTTCTTTATGGACATAAGGTGGTCTTTAATTGCCGGAAGATTGCCAGGAAGAACTGCCAATGATGTGAAGAACTATTGGAATACTTATCAAAGGAAAAAGGATCAAAAGACGGCTTCATACGCAAAGCAACTGAAAGTTAAATCTCAAGAAAATACAAAAGCCACCACAATTGTAAGACCTCGACCACGAACCTTCATCAAAAGGTTCAATTTTACGGAGAGATATGAAAATATAGAGCATAATCATTCAGAAATGAGTTATACCAGTTCTTTACCAACAGCACCACCACAGACTCTACAATTAGAAAATGTAACTGATTGGTGGAAAGATTTCGCAGAAGACAGTACAGAGAGCATTGATAGAACAATGTGTTCTGGTCTTATTGGTTTGGAGGATCATGACTTCTTCACAAACTTTTGGGTCGAAGATACGGTACAATCGGCAAGCAATGATCTAGTCAACATCTCCTACGTATGACTTCTTTAAGACTTCCTGGTGCATATTAGTAGTTGCACTTCTTTTTCTTTTTTCTTTTTTCCTCTTTATTTGGTTTCTCGTATGTATCTGGTTGTGCACTAGCTACTAGTAGGTTTCTATTGTATTTCGGTTAAATAAGAGATTGCACAAATACGTACGTAGATGGAGTAATATATATGGTTTTGCAGCTGAGGAGTTTATACGTCGATCTTCAGTGAGAAGAAGCCTAATTCATACAATACACGATGTAATCGATCTTCAAAAACCTCGAGACACACATATATGATTCAGTTAGGGCATGCATGTAGGTCAGTGCAGCTGGGTTCAAT

**Appendix S2. A 2046bp fragment (*fvemyb10-*Ter^myb10^) across *fvemyb10* genomic DNA and terminator of white-fruit accession YW5AF7.**

ATGGAGGGTTATTTCGGTGTGAGAAAAGGTGCATCGACTAAAGAGGAAGATGAACTTCTGAAACAGTTCATCGAAATTCATGGAGAAGGCAAATGGCATCATGTTCCTCTCAAATCAGGTAGTTAATCTAAGATTACGTACATGCTCTCACTGGAAAGTTTAGACTGCTGCTTATATGTATGTTTACGTCTGTACTTGCAGGCTTAAACAGATGCAGGAAGAGCTGTAGACTGAGATGGCTGAATTATTTGAAGCCGAATATCAAGAGAGGAGAGTTTGCAGAGGATGAAGTTGATTTGATCATCAGGCTTCATAAGCTTCTAGGAAACAGGTAATTAGAGCATCTGATATGTTCGTTTCTGCTTAATTTCTCAGTTACTGTTCTCTCAGGTAAAGCATGCGTTATTTCTTCTGGAATTCCAAAGCTAACTATATATATATATATATATATATATATATATTTACATATGTTTTCCTTGAATAATAAATATCTAGAGTGGTGGTCATTTCTGCTACTTTTACGCTTTTAATTAGCCCATGCATCCTCCTTTCATATCATGCGTACATTCCTTCATACAATCATACCCACTTACTTTCAGTGTAGGGAACTCCTTTAGCTGAAGTATTGGTGCTTATTATTGCCTGATGGCTACGGCTTATGCTTGCCTAGCTAGCTATCCATGAACTAAACATCAAAATGTCTACATAGCCTTTTAACGAAATTTATCATGAATTAATTACAAATTATTTGAACTAAAAAGATTCTTTTATAATTTCATAACTGACTGACAGATGTTTATATATAATATATATACACGTGTGATGTGAAGAACTATTGGAATATATAATATAGTGAGGATTTTGCTACTGTGTTTGATGCACGCTAAATTAAAACAAAAGAAGACAAATGTATGATCTTGTTCTTCTTCTTGTAATCCGTAAAAATTGGATTTTGTTTTCCAGATTTTAAAATGTGATTTTTCTTTCTTTTCTTTTAATTTGATTTGTTATTAAATTAGGTAATATCACGTCACTTGGCACATCAATTAGTAATATGTTGACATCATATTTAGGATTGATCTAGACCAATCATTGTTTTAAAATCTAATGGCTAACAATTAGTGTCAAAATTTGTGTCAAATTTAATGTCCCTTAATGTCCCTTGAACCGGACCCTATATGACATACATTTAATATTGTAGAAATTGCCTTGCATCTGTTAAGTTTCTAATGTGTTTCTTTTCTTTATGGACATAAGGTGGTCTTTAATTGCCGGAAGATTGCCAGGAAGAACTGCCAATGATGTGAAGAACTATTGGAATACTTATCAAAGGAAAAAGGATCAAAAGACGGCTTCATACGCAAAGCAACTGAAAGTTAAATCTCAAGAAAATACAAAAGCCACCACAATTGTAAGACCTCGACCACGAACCTTCATCAAAAGGTTCAATTTTACGGAGAGATATGAAAATATAGAGCATAATCATTCAGAAATGAGTTATACCAGTTCTTTACCAACAGCACCACCACAGACTCTACAATTAGAAAATGTAACTGATTGGTGGAAAGATTTCGCAGAAGACAGTACAGAGAGCATTGATAGAACAATGTGTTCTGGTCTTATTGGTTTGGAGGATCATGACTTCTTCACAAACTTTTGGGTCGAAGATACGGTACAATCGGCAAGCAATGATCTAGTCAACATCTCCTACGTATGACTTCTTTAAGACTTCCTGGTGCATATTAGTAGTTGCACTTCTTTTTCTTTTTTCTTTTTTCCTCTTTATTTGGTTTCTCGTATGTATCTGGTTGTGCACTAGCTACTAGTAGGTTTCTATTGTATTTCGGTTAAATAAGAGATTGCACAAATACGTACGTAGATGGAGTAATATATATGGTTTTGCAGCTGAGGAGTTTATACGTCGATCTTCAGTGAGAAGAAGCCTAATTCATACAATACACGATGTAATCGATCTTCAAAAACCTCGAGACACACATATATGATTCAGTTAGGGCATGCATGTAGGTCAGTGCAGCTGGGTTCAAT

**Appendix S3. Pro1: a 1475bp promoter fragment upstream of FvH4_2g26970 (gene1).**

GCATGGCTTCACACCAATAACACTACCATGTAGAAAGCCGCCGCCATCATAGTATGACATTGTAGAGAGTATTTCATGATTTATATCGGTGCAATTTAGTCTTTAAATTGTGTAGCTCGGTTCATTGTGTATTTAGAAATATCATTATATACAAGCGTTTCACGTTACAAAGTTCGTTGAAAACAATATTACGAAGATATGTTATGTATGTTTAGAAAGACCATGCCAGATCACTATGTCGAAAAGATTGAGCTTGGATGTATCTTGATTTTACACATTTTAAGCTGGATATTGTAATGTTCACCTTGGTCCTTTGCAAATGCATTATTTCCCAAAAGACAAGGTATTGTAAAATAAAAGTTATGTAGCTGGGCTCGGACGGTCCTTGTTCTCTTGGTAAGCCCAAAAGAAAAAGAATCTCGGTTCGAACCGGGTCATAATTGTGGGTAGATGCTTTCGGGTCATAATTCTGGGTAGATGCTTGTATGCCAGAGAAACACTCTCTTTCTCAAGCGTACAAAATGTGTCCAAAATTTTACGTGGCTTGCTCATTGATTTTGTGTAAATTTAAAGATAGCAAAATTATACAGTGGCTTATATTGTGCCACACAACACATCGAAGTATCAAGAAGCAATTGTTAGTGTTTGTGTTCAAATACGAACTCATTATTTGAACTGTCTTTTTGTTACCTTTTGCATCAACTTGTATTTTTGCAGGACTGGTTTTGAATCTGGGTATGTCATGTGTGGTTCACATCTCCCAACACTTGTGTGGACTTACTCATCACTGTGGTCTGAATGAGACCCAAGAGAAAGTTCCAAGTTAGGACATGTGTTTCTGTAAGTATTCCAGGTTCATTGCTGTAGAATTTCTTTGTTTAAGTAACCATGTTTACTTCTCTTGAATTCATTCCAAGATACGACAGACAACAAATGTTCTATAAAATGCCTTAAGAGACAAACAAAATCGTGTCTTATTTTGACGATCGGTGAATTAATGGTAAGATATTTCTACTGCTGGTGTTAGGAACCTGTGTTTCACATCATGTTTGGCCTTATGAAACAGAATACGCTTGATAGTAGAATCACATTACTCCTAGTACACTATGCAACAATTACATTGAATGTTCTGTGATATTTTATGGTTCTCAAGCTTTTGAAAATAGCTAGGTTGGTCTTACTTGACCAGTTAAGTCTTTACATGTGTCATTTTGTATGCAAGCTCTTATGCAATTTGAGGTTATACACTGGGGTAGGCTCCAACATTATCTTCAACATCACAAAGTGCACATACATAGACGTGGGCATTCTAATTGCCCACCAACTAGCTCACACACCATATCCACAATCTCTCAGTAAGATTGAAACTACTTGTATCACACACACCTCTAGTTCCCTCCTTAAATTGGGTTTGTGAGAAAACATAGTAGTCTACTAGTCTTCTGCAAGAGCAATTAAAACCCCTCAGATCAACA**ATG...**

**Appendix S4. Pro2: a 1060bp promoter fragment upstream of FvH4_7g22910 (gene2).**

CTTGCTTGTCCTTTGTACGCATAACAACTGATCGATTCGTATTATACATTTTACTTGAGTACTAATGAGAAAATTGTATTTGAGTTAAAAAAAAAAGGTATATAGCAAATTTCAAGGTGCAACTAGAACCTCCTAATTTGTATGGATAAGGTGCAGGAAAAAAAATTCGAGAAAAAAAAACATATAGTATTGACATAATTGAACATGTTTTGGCATGCTTTTGTCTTCCACGTTCCCAAGTTTCAATCGTGTAAACTATGCATATCGCCCTAGCTTTTTCATCAGACTGTCATGATACCAGTAATCTCAGTATGGGATACAATTTGGGCCTGATTTTTTATTTGAGGCTACAAGTGAAAAGGGATCTTGGGCTTCAATGAGCTAGGAAAAATGGATATATGCAGACCCGGATCTTTGACCCGGATTATACTGATTATGAACCAGCACAATTAGTCTTTTCCAGCACACACACGTCTGTAAATTATTGTATTTTTGTATAAATCCAAGAAAAAACAAAGAAATGAATATTGTTTCCAAAAATATTGTATTTTTGTTCAAATCCAAGAACAAACAGATACTGAATATCGTTGCCAATCACAAACATTTTATTCACCTCATCTCCAACTTTATATTTTCCTTCTCCCTTAATTCTATAGAAAAAACCAAAAAAGACCCACAAATAATGGAGATGGTTTTCTTCCGAAGTCTTCTATTCTCAAATCTGTGGTACTAACTTACCCTAACATCTGGTGGAGAAGATAATCCCTTGAAAACAAACCTCAAAACAAACCAATTAATGGAGACTGATAGTTGCATGCACTCTCCCTTCGCCACGTGGACGCAAGCCAATCTTGATTCTATGGTGGTGAGCAACTGAGTAAGCTAATCCTCAGGATGTGAATTTCTGTTCACTAGCTCAAACACAACGACTATAAGTTCAAACCCAGTTCCTCATCACAGACCACTCGATCTCTGCTTTTTGCTTGCAGGCAACAGTTGAGCTCCTCTTTGGTCCTCATTTTTAATATTACATCACATCCCTTCCGATCCTCCTCCGACA**ATG...**

**Appendix S5. Pro3: a 1352bp promoter fragment upstream of FvH4_6g38450 (gene3).**

TTGTTCAGCTTCCATTCCGTGGATGGAAAATTCTAGTATACCATATGGTATAACATACCTCACAAAATATGTGGCTATCATTGATTGAAAAAACAACTAAATGACCATGTAATGACTATTTACCCCTTCAAAAGCCCACATAGGCATGTCATACCATATGGTATGATAAACAAACCCTCCGTGGATATATTTGTTAAAGATCCTTGAATCATTTCCCCCAAAAAAGAAAAAGAAAAAAAAAGGAGATCCTTGAATCAGAGGTTATATAGGCTTTGATGAATGTCGTATATTGCTCGTCATTTTCTTGATGCATCTAATTAAGTACGTAGTTGGAGAAAACAAGATGCCAGTGAATCTAATACCAAGATTTCTTGCAGGGCTGCTTAATATCAAGTTATCAACTATTGTGAGTTACTAAAATGATTGAGTTCGTATGTGGGTGAAGATTTCACATGGAAATTTTGATACAGAGATATGTAATTATGCAAACAGATCCGTTCATATATTCCTTTCCATCGACAAACAAGTTACATCAACACAGGCACACTTGAAAGAAATCCCATTTACAACAGTTGCGCACACATGATTAAATCGATCACTTCACTCCAAAACTTTCAAACCTCACTTTCCGGGAACAAAGTTTGTGGCATATGACCAAGCGTTGTTGTTAACAGGGTCAGCCAAGTGATCAGCTAGGTTCTCAATCGGTCCCTTTCCGGTAACAATAGCTTGCACGAAGAATCCGAACATGGAAAACATAGCCAGTCTTCCGTTCTTGAGCTCCTTCACCTTAAGCTCAGCAAAAGCCTCAGGATCGTCTGCAAGCCCTAAAGGGTCAAAGCTCCCACCGGGGTACAACGGGTCTACTACCTCACCGAGAGGACCTCCTGCAATTCTGTAGCCTTCAACAGCACCCATCAAGATCACTTGTGTTGCCCAGATTGCCAAAATGCTTTGAGCGTGGACCAAGCTAGGGTTTCCCAAGTAGTCTAGTCCACCCTCACTGAAGATTTGAGCTCCAGCCTTGAACCAAACAGCTTCGCCGAACTTGACTCCATTACGAGCCAAGAGTTCAGGGAAAACACAGCCAAGGGCACCTAGCATGGCCCATCTGGAATGGATGACTTCGAGCTCACGGTTCTTGGCAAAGGTCTCAGGATCAGCTGAGAGCCCAGCTGTGTCCCAGCCGTAGTCACCGGGAAACTCTCCGGTGAGGTAAGACGGGGCTTCGCCGGAGAATGGTCCCAAGTACTTGACACGGTCAGGACCGTACCACGGGCTGACGGATGACACATTCTTTGACTTGGCTGCGGTTTTGCGCATGGTAACTCGGCCCTCACCGAGGAGGTTAG**ATG...**

**Appendix S6. Pro4: a 786bp promoter fragment upstream of FvH4_5g25760 (gene4).**

GATTACACTGATAGATTTAGCCTTTTTTCTCCTCAATAATAAGGGTAGTAATAAGGGTAGTTATCTTCTTTCAATTTGAGTGACCTTACTTTGGTGGATGTAATTGTACCTCTAATATTTGGGCAACATTAAATACCGTATTTCTTTAAGCTGCAGTGTAGCCTATAACAGATGTCACAGATCTGTCATGATATATAGGTATGAATAGTCTACTTTTTCCTTCTTTCTAGAAAATTAATTCTTAGCCCTAGTTATAAAACATTGAACAGGTGTGACTCGAGTGAATTTGTGCTCAGTTCAATGTCAGGCTCGCTGAAGAATCGGTGCTTTGTGGTGGAGCTGCTTCAAGTTTGAGACTTAAACATGTTGTCTGTGTCATGAAATGTGGGAGATGTTCTCATTGAGTTGAAGCAGTTTGCACCATCCACCATGTTTTCATGTGAGCCAGTAATCTCTGCTTATGCAAAACCTCTTGGATTTGGATTGGATAGGTCACCATTGAATATGTGACACTGTCTTCGTAACCTCTTAATGTAATTTGACATCCCCATTTGGAAGGACATTCCAGGAAAGAAGAATGAGGGAATCTTCTGTGGTTCATACTTTCGAACCCCATACAGAACATGATCTTATCTCATATATCTACTTCCTATACTATGCTAACCACAGGTTCCTATAATTCAACAGCCACCATAACTTAAAAAAAGGTAACGTTGAAGATTAAAGATCCAAATTGTAACTATATATATAGCATCCAAGTCACTCATCAAGCTTTAGTTGCAAACC**ATG...**

**Appendix S7. The promoter sequence of *FveU6-2*.**

CTTCCAGAGTGGGATCTCGCTTTGAAATTAAAATGCGGGACTCCTTATTTCACTCACTTTCAAGTCGTATTTCCACATCTCAACTGTACAATGTCTAAAACACAGTGTGTAGATCATTCATACAAAGTTCGTGTAAAGATTATTCAATGTATATTAACTTGGACAGGTGCCGTAAGCAGTCAAAATCTGTTCTGTTTTAAACCTTCATTCTTCTAGAATTGCACTTAAAGGGAGGACACGTGACGCGCACATGCTTTGGTAATATTAGTTCCAAGACTCCCACATCGAAGGAAGCGAAAACAGAAAGCTGTTCATATATGAGAACATTGCAGTATAGcTC

**Appendix S8. The coding sequence of *FvePDS* (FvH4_4g12690).**

ATGTCGCTCGGGGCTTCTGTCTCTGCCACCAACTTGACCCACCAAGCCAAAGTCATCAACACCCACCACCCACATAGCCCTGCGCTTTCCTTTCACGGCAGTGAGATTGTTGGCCGGAACTTGGGGTTTGTGTCCTCACATGCTAGTACTAGGCTGAGGAAGGGTGTTCAGCCTTTAAAGGTGGTTTGTGTGGATTATCCGAGACCCGAGCTTGACAATACTGTGAATTTTTTGGAAGCTGCGCTCTTATCTTCCTCCTTTAGATCCTCTCCTCGCCCAGCTAAACCCCTCAAGGTTGCCATTGCTGGTGCAGGTTTGGCTGGTTTGTCAACTGCAAAGTATTTGGCAGATGCAGGTCATAAACCTATCTTACTCGAAGCAAGAGATGTTTTAGGTGGAAAGAACTTGTTTGGGGAGCTAGGAATCGATGATCGCTTGCAGTGGAAGGAACACTCTATGATATTTGCAATGCCAAACAAGCCAGGAGAGTTCAGCCGGTTTGATTTCCCTGAAGTTCTGCCAGCACCCTTAAATGGAATATGGGCCATATTAAAGAACAATGAGATGCTGACATGGCCAGAAAAAGTGAAGTTTGCTATCGGACTTGTGCCTGCAATTCTTGGTGGACAAGCTTATGTTGAAGCTCAGGACGGCTTGACTGTAAAGGAGTGGATGAGAAAACAGGGGATACCTGATCGAGTAACTACTGAGGTGTTTATTGCCATGTCAAAGGCCCTTAACTTTATTAATCCTGACGAGCTCTCCATGCAATGTATATTGATTGCTTTGAATCGATTTCTTCAGGAGAAACACGGTTCCAAGATGGCTTTCCTTGATGGAAGTCCTCCCGAGAGACTCTGTTCACCAATAGTTGATCATATCCAGTCACTCGGTGGTGAAGTCCAACTTAATTCCCGATTACAGAAGATTGAGTTAAATAATGATGGAACTGTGAAGAGTTTTGTCCTAAATAATAACAGTGTGATTGAAGCGGATGCTTATGTATGTGCAGCTCCAGTTGACATCTTCAAGCTTCTAGTGCCTGAAAACTGGAAAGAGATTCCATATTTCAAGAAATTGGACAAACTAGTAGGAGTTCCAGTCATCAATGAGTATTACAATCCAAACCAGTCTATGCTGGAGTTGGTTTTTGCACCAGCTGAAGAATGGATTTCATGCAGCGATTCAGAAATTATTGATGCCACAATGGAAGAACTTGCAAAGCTGTTTCCCGATGAGATAGCTGCTGATCAAAGCAAAGCAAAGATCTTAAAGTACCATGTTGTTAAAACACCAAGGTCTGTGTACAAAACTATACCAGATTGTGAACCTTGCCGTCCATTGCAAAGATCTCCATTGGAGGGTTTCTATTTAACTGGAGACTATACAAAACAAAAATATTTAGCTTCTATGGAAGGTGCTGTTCTATCAGGGAAACTTTGTGCACAGGCAATTGTACAGGACTACGAATTGCTCGTTGCTCGTGGCCAGAAAAGGTTGGCTGAGGCAGCTGCTCGATGA

**Materials and methods**

**Plant materials and culture condition**

The 7th generation inbred lines of two Fragaria vesca (*F.vesca*) accessions, namely Yellow Wonder 5AF7 (YW5AF7, white-fruited) and Rüegen (Ru F7-4, red-fruited) were used in this study (Hawkins et al. 2016). All the *F.vesca* accessions, tobacco plants and transgenic lines were grown in a growth chamber (XUNON: PT-G850L5) at a temperature of 25℃/16h, light followed by 22℃/8h darkness with a relative humidity of 50%. Leaves of tobacco plants *Nicotiana benthamiana* were used for Agroinfiltration-mediated transient transformation. The leaves of YW5AF7 and Rüegen were sampled for genomic DNA (gDNA) extraction using a CTAB method (Oosumi et al. 2006).

**Selection of red-colored marker genes**

The gDNA sequences for three candidate red-reporter genes *FveMYB10*, *FveRAP* and *FveRAP-L2* were determined based on previous studies (Gao et al. 2020; Hawkins et al. 2016; Luo et al. 2018). A 2046 bp fragment across *FveMYB10* gDNA and terminator was PCR amplified from red-fruit accession Rüegen and denoted as *FveMYB10*-Ter^MYB10^. Similarly, a 1296 bp fragment across *FveRAP* gDNA along with terminator, and a 1039 bp fragment across *FveRAP-L2* gDNA and terminator were amplified from Rüegen and denoted as *FveRAP*-Ter^RAP^ and *FveRAP-L2*-Ter^RAP-L2^. All three DNA fragments were incorporated into over-expression vector JH23 via *Kpn*I and *Pac*I through homologous recombination (HR) using the ClonExpress Ultra One Step Cloning Kit (Vazyme) (Supplementary Table S1), respectively, resulting in AtUBQ:*FveMYB10*-Ter^MYB10^ (*FveMYB10*-OE), AtUBQ:*FveRAP*-Ter^RAP^ (*FveRAP*-OE) and AtUBQ:*FveRAP-L2*-Ter^RAP-L2^ (*FveRAP-L2*-OE). Three constructs were transfected into YW5AF7 calli through the *Agrobacterium*-mediated gene transformation method. Non-infiltrated plants were used as a negative control.

**Expression pattern of genes driven by four candidate promoters**

The tissue-specific gene expression pattern of four candidate genes were determined by RNA-seq database and visualized in BAR (<http://bar.utoronto.ca/efp_strawberry/cgi-bin/efpWeb.cgi?dataSource=Developmental_Map_Strawberry_Flower_and_Fruit>). YW5AF7 young tissues at different stages (callus-stage1, callus-stage2, young seedling and plantlet) were sampled for RNA extraction using a Polysaccharide Polyphenol Plant Total RNA Extraction Kit (TIANGEN, DP441) according to the manufacturer’s instructions. One microgram of total RNA was used for cDNA synthesis with a SPARKscript II RT Plus Kit (With gDNA Eraser) (Shandong Sparkjade Biotechnology Co., Ltd., AG0304-B). The cDNA product was diluted 10 times, and 1μL was used as a template in RT-qPCR using QuantStudio 5 Systems (Applied Biosystems, Foster City, CA, USA) along with a 2 × SYBR Green qPCR Mix (With ROX) (Shandong Sparkjade Biotechnology Co., Ltd., AH0104-B). The measurements of gene expression by RT-qPCR were performed in triplicate, and the mean of all these values for each gene was analyzed and normalized using the 2^−△△CT^ method. For all RT-qPCR reactions, FvH4_3g35181 (Histone protein) was used as the housekeeping gene. RT-qPCR primers are listed in Supplementary Table S1.

**Validation of the activity of candidate promoters**

The promoter sequences for these four candidate genes (Pro1: a 1475 bp promoter fragment upstream of FvH4_2g26970 (gene1); Pro2: a 1060 bp promoter fragment upstream of FvH4_7g22910 (gene2); Pro3: a 1352 bp promoter fragment upstream of FvH4_6g38450 (gene3) and Pro4: a 786 bp promoter fragment upstream of FvH4_5g25760 (gene4)) (Supplementary Table S2) were PCR-amplified with specific primers (Supplementary Table S1) from YW5AF7 young leaves and cloned into *GUS*-containing vector DX2181G through HR at *Hind*III-*Bam*HI sites, respectively, and denoted as Pro1:*GUS*, Pro2:*GUS*, Pro3:*GUS* and Pro4:*GUS*. These *GUS* constructs were transient expressed in *Nicotiana benthamiana* leaves and transfected into YW5AF7 calli through *Agrobacterium*-mediated gene transformation. *GUS* staining was performed as described previously (Ren et al. 2021).

**Vector constructions for CRISPR/Cas9 and genetic transformation**

The Cas9-containing destination vector JH19 showing high genome editing efficiency in diploid strawberry YW5AF7 (Zhou et al. 2018) was selected as a backbone via replacing At35S:3GFP cassette by red-reporter cassettes through *Kpn*I-*Asc*I digestion. Basically, the 1475 bp fragment of Pro1 and 2046 bp fragment of *FveMYB10*-Ter^MYB10^ were incorporated into construct JH19 through homologous recombination (HR) using the ClonExpress Ultra One Step Cloning Kit (Vazyme) at *Kpn*I-*Asc*I sites, resulting in AtUBQ:Cas9-Ter^ocs^-Pro1:*FveMYB10*-Ter^MYB10^ (JH27). Similarly, the 1060 bp fragment of Pro2 and 2046 bp fragment of *FveMYB10*-Ter^MYB10^ were incorporated into JH19 through same method, resulting in AtUBQ:Cas9-Ter^ocs^-Pro2:*FveMYB10*-Ter^MYB10^ (JH28). In the same way, the 786 bp fragment of Pro4 and 2046 bp fragment of *FveMYB10*-Ter^MYB10^ were also incorporated into JH19, resulting in AtUBQ:Cas9-Ter^ocs^-Pro4:*FveMYB10*-Ter^MYB10^ (JH29). As a control, the 1060 bp fragment of Pro2 and 2046 bp fragment of *fvemyb10*-Ter^myb10^, which was PCR amplified from white-fruit accession YW5AF7, were also incorporated into JH19, resulting in AtUBQ:Cas9-Ter^ocs^-Pro2:*fvemyb10*-Ter^myb10^ (JH30). All primers used in this study are listed in Supplementary Table S1.

*FvePDS* was selected as a marker gene determining genome editing efficiency of our system. Two seed guide RNA sequences (gRNA1: GCTAGTACTAGGCTGAGGAA and gRNA2: GGACTTGTGCCTGCAATTCT) (Supplementary Table S1) targeting *FvePDS* were inserted into the entry vector JH4 and incorporated into all above CRISPR constructs (JH27, JH28, JH29 and JH30) via gateway cloning, resulting in destination constructs: AtUBQ:Cas9-Ter^ocs^-Pro1:*FveMYB10*-Ter^MYB10^-sgRNA(*PDS*) (JH27-*PDS*), AtUBQ:Cas9-Ter^ocs^-Pro2:*FveMYB10*-Ter^MYB10^-sgRNA(*PDS*) (JH28-*PDS*), AtUBQ:Cas9-Ter^ocs^-Pro4:*FveMYB10*-Ter^MYB10^-sgRNA(*PDS*) (JH29-*PDS*), as well as the control construct AtUBQ:Cas9-Ter^ocs^-Pro2:*fvemyb10*-Ter^myb10^-sgRNA(*PDS*) (JH30-*PDS*). All four constructs were transfected into *Agrobacterium* strain GV3101 and transformed into white-fruit accession YW5AF7 calli and *Rubus idaeus* ‘Joan J’ calli following the protocol as described previously (Zhou et al. 2021). The red pigments of each transgenic lines were visualized at different stages (callus, young seedling, plantlet and mature plant) and recorded by ZEISS SteREO Discovery V20 stereomicroscope.

**Genotyping and mutation type detection**

Around 60 transformed calli were randomly selected from each construct at callus-stage 2, for testing the sensitivity of our native red-color reporter system as well as genome editing efficiency. For JH27-*PDS*, 31 calli presented red color and 28 calli did not, for JH28-*PDS*, 32 red calli and 31 non-red calli were identified. Genomic DNA was extracted from the selected calli using a Plant Genomic DNA Kit (TIANGEN, DP305) and T-DNA insertions were identified using the primers Cas9-F2 and Cas9-R4 (Zhou et al. 2021) (Supplementary Table S1). Moreover, the primers Pro2-seq and *FveMYB10*-seq were used to detect the presence of *FveMYB10* in the T-DNA insertion portion of JH28-*PDS* transgenic lines (Supplementary Table S1). 2 × Taq Master Mix (Dye Plus) (Vazyme) was used for PCR reactions.

To identify the mutation events of the target gene, 2 × Phanta Max Master Mix (Dye Plus) (Vazyme) was used to amplify the two sgRNA sites of *FvePDS* gene using the aforementioned gDNA as a template. We detected different mutation types in all transgenic lines by using primer pairs as shown in Supplementary Table S1 (*FvePDS*-F1 and *FvePDS*-R1 for sgRNA 1, *FvePDS*-F2 and *FvePDS*-R2 for sgRNA 2). The Sanger sequencing was performed by Qingdao Weilai Biotechnology Co., Ltd., and the sequencing results were decoded using the online CRISPR/Cas9 mutagenesis decode tool (http://skl.scau.edu.cn/dsdecode/). The total anthocyanins contents in T_0_ leaves of JH27-*PDS,* JH28-*PDS,* JH29-*PDS* and JH30-*PDS* transgenic plants were measured following previous research (Luo et al. 2018), and wild type of YW5AF7 plants were used as control. Three independent biological replicates and three technical replicates were measured in this assay.

The potential *FvePDS* off-target sites were determined via CRISPRdirect (https://crispr.dbcls.jp/), and Fvb1:15026687-15026697 off-target site 1 and [Fvb6:29643319-29643329](https://www.rosaceae.org/jbrowse/?data=data/fragaria/fvesca_v2.0.a1&loc=Fvb6:29643319..29643329&tracks=DNA,genes,transcripts) off-target site 2 were chosen for analysis. The genomic DNA sequences across the off-target site 1 and off-target site 2 were amplified with primer pairs PDS-off-target-1F/PDS-off-target-1R and PDS-off-target-2F/PDS-off-target-2R (Supplementary Table S1) and then sequenced.

**Gene & Accession Numbers**

The sequence data (Supplementary Table S2) for the *F.vesca* genes can be found in GDR (www.Rosaceae.org) using Sequence Retrieval tool using gene IDs (genome version 2.0.a2, version 4.0.a2): gene 1 (gene11180, FvH4_2g26970), gene 2 (gene26333, FvH4_7g22910), gene 3 (gene28726, FvH4_6g38450), gene 4 (gene31500, FvH4_5g25760), *FveMYB10* (gene31413, FvH4_1g22020), *FveRAP* (gene31672, FvH4_1g27460), *FveRAP-L2* (gene08595, NA) and *FvePDS* (gene16877, FvH4_4g12690).

**Abbreviations**

GUS: β-glucuronidase

NVSR: Native Visual Screening Reporter

CRISPR/Cas9: Clustered regularly interspaced short palindromic repeats/CRISPR-associated protein 9

*PDS*: Phytoene Desaturase

sgRNA: single guide RNA

gDNA: genomic DNA

RAP: reduced anthocyanins in petioles

GFP: Green fluorescent protein

HR: homologous recombination

**References**

Gao Q, Luo H, Li Y, Liu Z, Kang C. Genetic modulation of RAP alters fruit coloration in both wild and cultivated strawberry. Plant Biotechnol J. 2020;18:1550-61. <https://doi.org/10.1111/pbi.13317>.

Hawkins C, Caruana J, Schiksnis E, Liu Z. Genome-scale DNA variant analysis and functional validation of a SNP underlying yellow fruit color in wild strawberry. Sci Rep. 2016;6:29017. <https://doi.org/10.1038/srep29017>.

Luo H, Dai C, Li Y, Feng J, Liu Z, Kang C. Reduced anthocyanins in petioles codes for a GST anthocyanin transporter that is essential for the foliage and fruit coloration in strawberry. J Exp Bot. 2018;69:2595-608. <https://doi.org/10.1093/jxb/ery096>.

Oosumi T, Gruszewski HA, Blischak LA, Baxter AJ, Wadl PA, Shuman JL, et al. High-efficiency transformation of the diploid strawberry (Fragaria vesca) for functional genomics. Planta. 2006;223:1219-30. <https://doi.org/10.1007/s00425-005-0170-3>.

Ren C, Liu Y, Guo Y, Duan W, Fan P, Li S, et al. Optimizing the CRISPR/Cas9 system for genome editing in grape by using grape promoters. Hortic Res. 2021;8:52. <https://doi.org/10.1038/s41438-021-00489-z>.

Zhou J, Sittmann J, Guo L, Xiao Y, Huang X, Pulapaka A, et al. Gibberellin and auxin signaling genes RGA1 and ARF8 repress accessory fruit initiation in diploid strawberry. Plant Physiol. 2021;185:1059-75. <https://doi.org/10.1093/plphys/kiaa087>.

Zhou J, Wang G, Liu Z. Efficient genome editing of wild strawberry genes, vector development and validation. Plant Biotechnol J. 2018;16:1868-77. <https://doi.org/10.1111/pbi.12922>.
